# Autism Identification Based on the Intelligent Analysis of Facial Behaviors: An Approach Combining Coarse- and Fine-Grained Analysis

**DOI:** 10.3390/children11111306

**Published:** 2024-10-28

**Authors:** Jingying Chen, Chang Chen, Ruyi Xu, Leyuan Liu

**Affiliations:** 1 Faculty of Artificial Intelligence in Education, Central China Normal University, Wuhan 430079, China; chenjy@mail.ccnu.edu.cn (J.C.); c-chen2020@mails.ccnu.edu.cn (C.C.); lyliu@ccnu.edu.cn (L.L.); 2 Computer Science and Artificial Intelligence School, Wuhan University of Technology, Wuhan 430070, China

**Keywords:** autism identification, head pose, facial expression Intensity and types, LSTM, feature-level attention mechanism

## Abstract

Background: Facial behavior has emerged as a crucial biomarker for autism identification. However, heterogeneity among individuals with autism poses a significant obstacle to traditional feature extraction methods, which often lack the necessary discriminative power. While deep-learning methods hold promise, they are often criticized for their lack of interpretability. Methods: To address these challenges, we developed an innovative facial behavior characterization model that integrates coarse- and fine-grained analyses for intelligent autism identification. The coarse-grained analysis provides a holistic view by computing statistical measures related to facial behavior characteristics. In contrast, the fine-grained component uncovers subtle temporal fluctuations by employing a long short-term memory (LSTM) model to capture the temporal dynamics of head pose, facial expression intensity, and expression types. To fully harness the strengths of both analyses, we implemented a feature-level attention mechanism. This not only enhances the model’s interpretability but also provides valuable insights by highlighting the most influential features through attention weights. Results: Upon evaluation using three-fold cross-validation on a self-constructed autism dataset, our integrated approach achieved an average recognition accuracy of 88.74%, surpassing the standalone coarse-grained analysis by 8.49%. Conclusions: This experimental result underscores the improved generalizability of facial behavior features and effectively mitigates the complexities stemming from the pronounced intragroup variability of those with autism, thereby contributing to more accurate and interpretable autism identification.

## 1. Introduction

Autism spectrum disorder (ASD) is a neurodevelopmental disorder characterized by core symptoms, including difficulties in social interaction, communication impairments, and the exhibition of repetitive, stereotyped behaviors. In China, the number of individuals affected by ASD is increasing annually, with some regions reporting a prevalence rate as high as 0.7% [1]. According to recent data released by the Centers for Disease Control and Prevention (CDC) in the United States, the prevalence rate of autism spectrum disorder (ASD) in the United States in 2020 was approximately 2.7%, which is higher than the global average prevalence rate of 62 per 10,000 reported by the World Health Organization (WHO) [2]. Many autistic individuals struggle to manage their core symptoms, which can hinder their ability to engage fully in social activities. Currently, educational interventions serve as the cornerstone of ASD rehabilitation. To maximize the effectiveness of these interventions, accurate early identification of ASD in the field of special education is crucial. Nevertheless, traditional methods that rely on assessment with observation scales can be professional yet time-consuming, highlighting the need for innovative, intelligent approaches to ASD identification [3]. Taking into consideration that a lack of empathy is a crucial factor [4] that exacerbates difficulties in social engagement among individuals with ASD and given that facial expressions [5] act as a tangible representation of underlying emotional states, this research endeavored to utilize facial behaviors as biomarkers for the identification of ASD.

By examining previous research on facial expression behaviors in autism, we highlighted three distinct characteristics of facial behaviors that have been associated with ASD: (1) a tendency to exhibit neutral or low-intensity facial expressions in social situations, (2) poor social congruency in communicating emotions through facial expressions, and (3) difficulty in facial expressions being accepted or understood by others. Each of these behaviors is described in more detail in the following paragraphs.

A tendency to exhibit neutral or low-intensity facial expressions in social situations. According to research conducted by Yirmiya et al. [6], it has been observed that during structured play scenarios, children with ASD tend to exhibit more neutral or bland facial expressions, whereas children with intellectual disabilities express more positive facial expressions. The authors emphasized that social engagement during structured play often fosters positive emotions among participants. The high prevalence of neutral expressions and the scarcity of positive emotions among children with ASD indicate a lower level of social engagement compared to children with intellectual disabilities. Moreover, Capps et al. [7] investigated the correlation between the proportion of positive facial expressions in children with ASD and their empathy abilities, revealing a significant positive association. This suggests that diminished social interaction among children with ASD may stem from empathy deficits. Other pertinent studies have arrived at similar conclusions. For instance, Trevisan et al. [8] examined the relationship between facial expression production and the characteristics of alexithymia. Their research found that spontaneous facial expressions triggered by emotional videos differed significantly between children with ASD and typically developing children. During emotional elicitation, children with ASD displayed neutral expressions for considerably longer durations compared to the control group. Notably, their study indicated that characteristics of alexithymia, rather than ASD characteristics, predicted variations in facial expression production among participants, suggesting that alexithymia may account for deficits in emotion recognition, empathy, and interoceptive accuracy among individuals with ASD.

Poor social congruency in communicating emotions through facial expressions. A study conducted by Dawson et al. [9] with 16 children with ASD, with an average age of 4.13 years, and 16 typically developing children, uncovered a notable difference in facial expressions during face-to-face interactions with their mothers. Children with ASD smiled and responded with facial expressions significantly less frequently than their typically developing peers. Furthermore, even when facial expressions were present, they often lacked eye contact. In another study by Mcgee [10], preschool activities in an inclusive kindergarten were observed, involving five children with ASD and five typically developing children. The research revealed that children with ASD and typically developing children displayed distinct facial expressions in various scenarios. For example, typically developing children tended to exhibit happy facial expressions when interacting with teachers or peers, whereas children with ASD showed happy expressions more often when playing alone. Additionally, typically developing children were more prone to displaying angry facial expressions in situations involving other children, whereas children with ASD were more likely to become angry in situations involving adults.

Difficulty in facial expressions being accepted or understood by others. The facial expressions displayed by children with ASD are often described as awkward, stiff, strange, or inconsistent. These expressions can be challenging for individuals in the general population to interpret accurately. Grossman et al. [11] examined the facial expression characteristics of high-functioning children with ASD during a story-retelling task. Their findings revealed that, compared to typically developing children, high-functioning children with ASD exhibited significantly more awkward facial expressions. Notably, this awkwardness was not readily apparent in static images but was evident in dynamic sequences. This underscores the importance of the dynamic transition of facial expressions in social interactions and the negative impact of awkward dynamic facial expressions on social engagement in ASD. Volker et al. [12] delved into the encoding abilities (emotion portrayal) of various facial expressions among high-functioning individuals with ASD. Their results indicated that high-functioning individuals with ASD had little difficulty encoding happy expressions, suggesting that happy expressions were the most easily portrayed. However, they struggled with encoding sad expressions and displayed significant strangeness compared to the control group. While differences in encoding other facial expressions were relatively minor, they generally exhibited difficulties encoding anger and fear while proficiently encoding disgust and surprise. A study conducted by Gordon et al. [13] focused on 17 children with ASD (average age: 10.76 years) and 17 typically developing children. It revealed that the quality of happy and angry expressions produced by children with ASD was lower than that of typically developing children. However, this difference could be significantly improved through play-based intervention training.

To intelligently describe the facial behavioral traits of autism, existing approaches simulate the scale evaluation process through manual feature extraction and statistical analysis [14]. Although such evaluation methods offer good interpretability, their limited representational capacity in manual features fails to adequately address the notable intraclass variability observed in autism. In contrast, leveraging deep-learning methods to extract adaptive features from data can overcome the issue of inadequate representation [15]. Deep learning has achieved significant breakthroughs in automatic facial expression analysis. By utilizing cascaded artificial neural networks, deep learning is able to extract rich and representative high-level semantics, enabling it to accurately capture subtle appearance changes induced by spontaneous facial expressions. This significantly enhances the accuracy of recognition of various facial behaviors. For instance, Li et al. proposed the Graph Convolutional Networks (GCNs)-based method FG-AGR [16], which achieved a recognition accuracy of 90.81% on the RAF-DB dataset. Similarly, Mao et al. proposed the method Poster++ [17], leveraging Vision Transformers (ViT) [18], and attained a higher recognition accuracy of 92.21% on the same dataset. These advancements in deep learning for facial expression analysis have paved the way for its application in autism identification. For example, Saranya et al. [15] incorporated Facial Action Coding System (FACS) features into a CNN-LSTM model for autism identification, achieving a recognition accuracy of 92% on the Kaggle Autistic Facial Datasets (KAFD-2020). Wang et al. [19] employed CNNs to extract multiple features, which were then fused and input into an LSTM for autism identification. This model, using only facial expression features, achieved recognition accuracy of 89.39% on the Ext-Dataset [20]. When eye movement features were also included, the accuracy increased to 98.54%. However, they often struggle to provide convincing interpretability. These limitations pose considerable barriers to the widespread adoption and effective deployment of intelligent identification systems.

To bridge this gap, we propose a facial behavior characterization model that integrates coarse- and fine-grained analytical approaches (Section 2). Following this integration, we undertake a detailed exploration of the algorithm’s implementation, offering a comprehensive understanding of its operational mechanisms and functionalities (Section 3). Finally, to ensure the validity and practicality of our proposed method, we tested its efficacy through a series of experiments while also convincingly demonstrating its interpretability. This ensures that our model is not only suitable for real-world applications but also comprehensible to a diverse range of stakeholders (Section 4).

In summary, the primary contributions of this paper are as follows:

1. Proposing a hybrid model of coarse- and fine-grained behavioral characterization. The coarse-grained analysis emulates a scale-based evaluation approach, enhancing interpretability, while the fine-grained analysis leverages long short-term memory (LSTM) networks to explore subtle temporal dynamics, thereby augmenting the discriminative power of the entire model;
2. Introducing an attention-based end-to-end neural network. This network is designed to integrate coarse- and fine-grained analytical features with a squeeze-and-excitation network (SENet). An attention mechanism was employed to illuminate the contributions of individual features in the decision-making process, offering valuable insights into the relative importance of different behavioral cues;
3. Experimental validation on a self-constructed dataset. We conducted extensive experiments on our self-constructed dataset, demonstrating the feasibility of leveraging facial behavior data to identify autism. Our findings indicate that the proposed fusion approach significantly outperformed a sole coarse-grained approach, substantiating its potential to improve the accuracy of autism identification.

## 2. Facial Behavior Characterization Model

**Figure 1:**
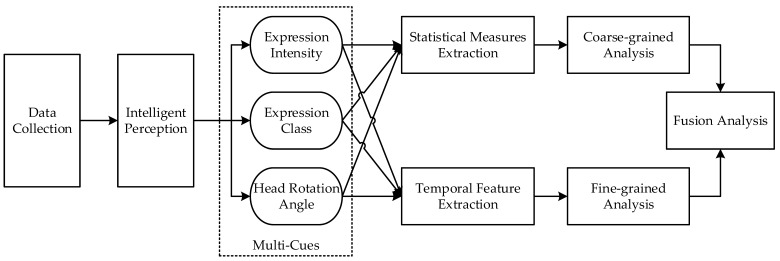
A combined coarse- and fine-grained facial behavior characterization model.

Due to the spectrum distribution of autism symptoms and notable individual differences, we propose a combined coarse- and fine-grained facial behavior characterization model to comprehensively describe the facial behavior characteristics of individuals with autism. As shown in Figure 1, this model uses optical cameras to capture facial data of the subjects and applies artificial intelligence algorithms to perceive facial expressions and head poses within the data, obtaining frame-by-frame analysis results in three dimensions: expression category, expression intensity, and head pose. Subsequently, statistical and temporal features were extracted from the three-dimensional temporal data for subsequent coarse- and fine-grained analyses. Specifically, statistical features include the average intensity of expressions, the occurrence rate of context-consistent expressions, and the range of head pose variations, whereas temporal features are extracted using the deep learning model LSTM. In this process, the expression categories and intensities reveal the individual’s emotional understanding and interaction capabilities when viewing specific emotional stimuli, while the head pose reflects the individual’s attention. It is important to note that when an individual’s attention is diverted, their emotional understanding and interaction processes are also interrupted. Therefore, estimating attention is crucial in emotion-related intelligence analysis.

In the proposed model, coarse-grained analysis mainly uses a binary approach (“yes” or “no”) to judge the behavior of individuals within a specific period. Specifically, coarse-grained analysis focuses on the following three aspects:

1. Does the individual’s attention deviate?
2. Has the individual’s facial expression reached a certain intensity level?
3. Does the individual display a specific facial expression (e.g., a happy expression)?

These three observations point to three issues in emotional understanding and interaction in autism: difficulty maintaining attention [21], weak facial expression intensity [6], and poor social consistency in emotional expression [10]. This analysis method is similar to traditional scale assessment methods [22], in which the presence or absence of described phenomena is judged, providing good interpretability.

Fine-grained analysis refers to further in-depth analysis of the temporal evolution characteristics of an individual’s facial behavior, assuming similar results in coarse-grained judgments. Specifically, fine-grained analysis focuses on the following three aspects:

1. Is the individual’s head rotation variation atypical? [23]
2. Are the changes in the intensity of the individual’s facial expressions atypical? [24]
3. Is the evolution of the categories of the individual’s facial expressions atypical? [25]

The goal of fine-grained analysis is to identify atypical head rotation [23] and facial expressions [24,25] in individuals with autism (i.e., those that are difficult for typical individuals to understand and accept). Given the significant variations observed in individuals with autism and the lack of clear, unified characterizations and definitions of atypical dynamic facial expressions, traditional scale assessment methods are often influenced by the subjective factors of the evaluators. In contrast, this research uses the facial expression intensity estimation method in [26], the expression recognition method in [27], and the head pose estimation method in [28] to automatically extract features from the data, effectively avoiding subjective interference and ensuring better objectivity and accuracy.

The relationship between coarse- and fine-grained analysis is as follows: the coarse-grained analysis forms the basis and premise of the fine-grained analysis. When coarse-grained analysis results can reveal atypical patterns, it is possible to prioritize separating easily distinguishable individuals, reducing the workload and difficulty of the fine-grained analysis. When coarse-grained analysis results fail to identify atypical patterns, fine-grained analysis is used for further differentiation. The fine-grained analysis complements and extends the coarse-grained analysis. The coarse-grained analysis only addresses the first and second characteristics of facial expressions in autism, as mentioned in Section 1, while the fine-grained analysis supplements the analysis of the third characteristic. Given their good complementarity, integrating the results of both coarse- and fine-grained analyses achieves a comprehensive characterization of the facial behavior of individuals with autism, thereby increasing the reliability of the autism assessment results.

## 3. Methods

This Section introduces our method for autism identification. It begins with an overview of the overall framework of the model, followed by a detailed focus on the implementation details of each key module.

### 3.1. Overview

The proposed method includes three main modules: coarse-grained analysis, fine-grained analysis, and fusion analysis. Before intelligent analysis, the collected facial expression sequences needed to be clipped according to the start time of the viewed video stimuli. These clipped video segments are converted into image sequences, which we refer to as “slices”. For each slice, the coarse-grained analysis involves calculating and analyzing statistics related to facial behavior features, while the fine-grained analysis captures temporal changes in head pose, facial expression intensity, and facial expression types using an LSTM model [29]. The fusion analysis combines these coarse-grained and fine-grained features using a feature-level attention mechanism in the squeeze-and-excitation network (SENet) approach [30].

### 3.2. Coarse-Grained Analysis

The input for coarse-grained analysis consists of facial expression intensity, facial expression category, and head pose estimation results within a slice, denoted as $I=\left\{I_{1},\ldots ,I_{K}\right\}$, $C=\left\{C_{1},\ldots ,C_{K}\right\}$, and $P=\left\{P_{1},\ldots ,P_{K}\right\}$, respectively, where *K* represents the number of slices (in the experiment, *K* was set to 6, corresponding to the six segments of emotional stimuli viewed by the subjects). Each slice is downsampled at equal time intervals to obtain the same length, denoted as *L* (in the experiment, *L* was set to 200). Given $k\in \left(1,\ldots ,K\right)$, $I_{k}\in R^{L\times 6}$ represents the facial expression intensity of six basic expressions (i.e., anger, disgust, fear, happiness, sadness, and surprise), with each element in $I_{k}$ ranging from $\left[0,1\right]$. $C_{k}\in R^{L\times 1}$ represents the vector of predicted categories for all frames in the *k*-th slice by the facial expression model, where the category label “happy” is encoded as 1, and all other facial expression categories are encoded as 0. $P_{k}\in R^{L\times 3}$ comes from the head pose estimation algorithm’s predicted three-dimensional Euler angles: pitch, roll, and yaw, with each dimension’s range being $\left[-\pi /2,\pi /2\right]$.

Coarse-grained analysis calculates different statistics for each of the above variables. Specifically, for each slice, the average facial expression intensity ${\bar{I}}_{k}$ is computed as follows:

(1) $${\bar{I}}_{k}=\frac{1}{L}\sum_{j\in \{1,\ldots ,L\}}max(I_{k}^{j}),$$

where $max\left(\cdot \right)$ calculates the maximum value of the input data, which is the maximum value among the outputs of the six facial expression intensity estimation models.

For each slice, the number of occurrences of happy expressions ${\bar{C}}_{k}$ is calculated as follows:

(2) $${\bar{C}}_{k}=\sum_{j\in \{1,\ldots ,L\}}C_{k}^{j}$$

For each slice, the range of head pose variations ${\bar{P}}_{k}$ is calculated as follows:

(3) $${\bar{P}}_{k}=max\left(P_{k}\right)-min\left(P_{k}\right),$$

where $max\left(\cdot \right)$ and min$\left(\cdot \right)$ designate the maximum and minimum values of the input data, respectively.

Discrimination rules are designed for the above statistics to derive binary features. Specifically, if the average facial expression intensity of the *k*-th slice is greater than the threshold $T_{1}$ (where $T_{1}$ is set to 0.3), the facial expression intensity is considered activated, denoted as 1; otherwise, it is denoted as 0. The specific mathematical formula is

(4) $${\overset{ˇ}{I}}_{k}=\left\{\begin{array}{cc} 1 & {\bar{I}}_{k}\geq T_{1}\\ 0 & {\bar{I}}_{k}<T_{1} \end{array}\right.$$

If the number of occurrences of happy expressions in the k-th slice is greater than the threshold $T_{2}$ (where $T_{2}$ is set to 10 to suppress potential short-term noise), the subject is considered to have displayed happy emotions, denoted as 1; otherwise, it is denoted as 0. The specific mathematical formula is

(5) $${\overset{ˇ}{C}}_{k}=\left\{\begin{array}{cc} 1 & {\bar{C}}_{k}\geq T_{2}\\ 0 & {\bar{C}}_{k}<T_{2} \end{array}\right.$$

If any Euler angle of the head pose in the k-th slice is greater than threshold $T_{3}$ (where $T_{3}$ is set to π/3), the individual’s attention is considered to have deviated, denoted as 0; otherwise, it is denoted as 1. The specific mathematical formula is

(6) $${\overset{ˇ}{P}}_{k}=\left\{\begin{array}{cc} 0 & max({\bar{P}}_{k})\geq T_{3}\\ 1 & max({\bar{P}}_{k})<T_{3} \end{array}\right.$$

Finally, the coarse-grained analysis result $O_{c}$ is obtained. It is represented as follows:

(7) $$O_{c}=[{\overset{ˇ}{I}}_{1},\ldots ,{\overset{ˇ}{I}}_{K},{\overset{ˇ}{C}}_{1},\ldots ,{\overset{ˇ}{C}}_{K},{\overset{ˇ}{P}}_{1},\ldots ,{\overset{ˇ}{P}}_{K}]\in R^{3K}$$

In the paper, K=6, resulting in $O_{c}$, outputting 18 feature values as the characteristics of the coarse-grained analysis.

### 3.3. Fine-Grained Analysis

**Figure 2:**
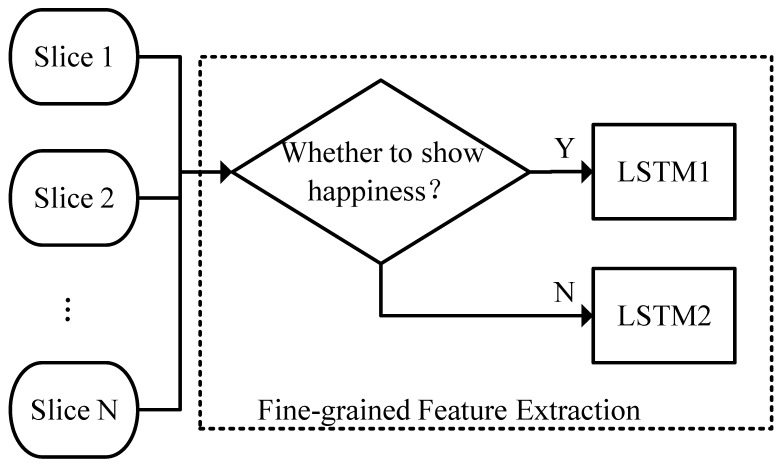
Fine-grained analysis.

The network structure for the fine-grained analysis is shown in Figure 2. The input for fine-grained analysis is slightly different from that for coarse-grained analysis. To describe the ambiguity of facial expressions in autism, this research uses the probability distribution predicted by the classifier as the fine-grained facial expression category feature, denoted as $C^{\prime }=\left\{C_{1}^{\prime },\ldots ,C_{K}^{\prime }\right\}$. Given $k\in \left(1,\ldots ,K\right),C_{k}^{\prime }\in R^{L\times c}$, where c represents the dimension of the classifier’s output probability distribution. In the experiment, a seven-class facial expression recognition model (six basic classes and a neutral expression) was used, so c=7. If the peak of the probability distribution tends toward a single category, it indicates low ambiguity of the facial expression; if the peak tends toward multiple categories or there is no clear peak, it indicates high ambiguity of the facial expression. For facial expression intensity features, only the prediction results of the happy expression intensity model are retained to eliminate redundant information, denoted as $I^{\prime }=\left\{I_{1}^{\prime },\ldots ,I_{K}^{\prime }\right\}\in R^{L\times 3}$, given $k\in \left(1,\ldots ,K\right)$, $I_{k}^{\prime }\in R^{L\times 1}$. The head pose features remain consistent with the input for coarse-grained analysis. By concatenating all the features, we obtain the fine-grained input $I_{f}={\left\{I_{f}^{k}\right\}}_{k=1}^{K}$:

(8) $$I_{f}^{k}=\left[I_{k}^{\prime },C_{k}^{\prime },P_{k}\right]\in R^{L\times d}$$

where d=1+7+3=11. That is to say, each image has a feature dimension of 11 for fine-grained analysis, comprising one intensity feature, seven expression features, and three head pose features.

For the *k*-th slice, its fine-grained features are input into the corresponding LSTM neural network based on the coarse-grained facial expression category analysis results to extract temporal feature $t^{k}$. The specific mathematical expression is

(9) $$t^{k}=\left\{\begin{array}{cc} LSTM1(I_{f}^{k}) & C_{k}=1\\ LSTM2(I_{f}^{k}) & C_{k}=0 \end{array}\right.,$$

where $LSTM1\left(\cdot \right)$ and LSTM2(·) are two LSTM networks with identical structures, but the trainable parameters of the network are obtained from different data. Specifically, the slices identified as “happy” by the coarse-grained analysis are used to train LSTM1, while the slices not identified as “happy” are used to train LSTM2. By dividing the data based on the coarse-grained analysis results and then inputting them into different LSTMs, the difficulty of the temporal feature extraction tasks is reduced, allowing a focus on the differences between individuals with autism and typically developing individuals under the premise of similar coarse-grained analysis results.

The extracted temporal features are projected through a linear layer to obtain the fine-grained feature indicators. The specific mathematical expression is

(10) $$O_{f}=\left[{LN}_{1}(t^{1}),\ldots ,{LN}_{K}(t^{K})\right]\in R^{K\times e}$$

where ${LN}_{k}\left(\cdot \right)$ reduces the feature dimension of the k-th output signal to e dimensions. In the experiment, we simply set e to 1.

### 3.4. Fusion Analysis

The fusion analysis module concatenates the results of the coarse-grained analysis and the fine-grained analysis into a single feature vector, which is then input into a simplified SENet to obtain feature weights and achieve feature recombination. Specifically, according to Formulas (7) and (10), we can obtain all the indicators that need to be analyzed in fusion O:

(11) $$O=[O_{c},O_{f}]\in R^{K\times (3+e)}$$

**Figure 3:**
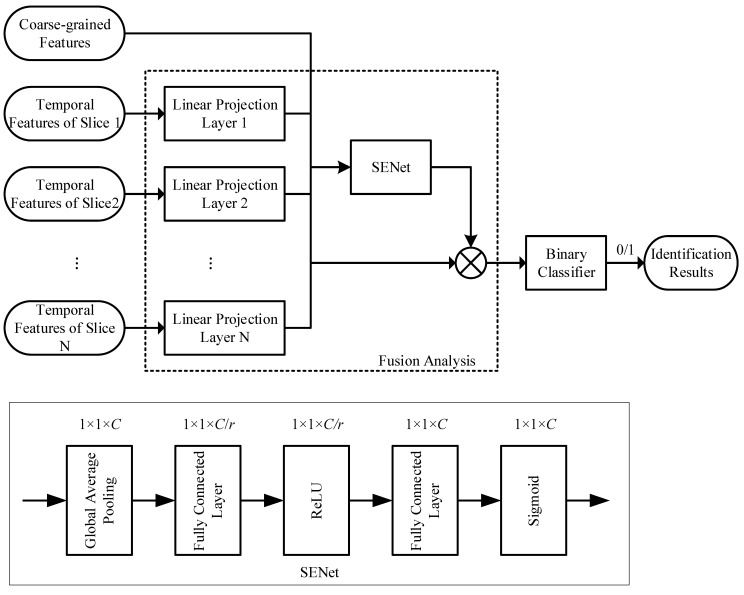
Feature fusion analysis with SENet.

SENet was proposed by Hu et al. [30] with the aim of extracting channel attention from convolutional neural networks and implementing the discarding or retaining of feature maps in different channels based on attention weights. Its network structure is shown in Figure 3.

In Figure 3, global pooling is used to compress a feature map of a convolutional neural network into a single feature value. The first fully connected layer compresses the feature values (the “squeeze”), reducing the original C feature channels to C/r dimensions. The second fully connected layer then restores the feature dimension back to C and “excites” (i.e., the excitation) the useful features. This compression and restoration process ensures that the attention weights of useful features approach 1, while the attention weights of useless features approach 0. Multiplying these attention weights by the corresponding feature channels retains the useful features and suppresses the useless ones. The entire network structure can be expressed by the following formula:

(12) $$A=\sigma \left(W_{2}\delta (W_{1}O)\right),$$

where σ(·) and δ(·) are the sigmoid and rectified linear unit (ReLU) activation functions, respectively, and $W_{1}$ and $W_{2}$ are the trainable parameters of the first and second fully connected layers, respectively. ***A***$\in R^{C}$ represents the attention weights of each channel.

The input features in this paper are K×(3+e) dimensions, where each dimension can be considered as a feature channel. Therefore, the first module (global pooling) in Figure 3 is not needed, and the input can be directly fed into the two fully connected layers of the SENet to compute the fusion weights. After obtaining the attention weights, the final output features are

(13) $$O^{’}=O\cdot A$$

The output features are passed through a binary classifier to obtain the final evaluation results.

## 4. Experiments

This Section describes the validation of the proposed intelligent autism assessment method, which combines coarse-grained and fine-grained analyses through comprehensive experiments. First, we introduce the self-constructed dataset and the details of the experimental setup. We then explain the effectiveness of each feature in the coarse-grained analysis through significance analysis. Next, we demonstrate the effectiveness of each module in the method through ablation studies. Finally, we further explain the interpretability of the method through attention visualization.

### 4.1. Dataset

Due to privacy concerns for individuals with autism, there are few publicly available autism emotion datasets in the existing research. In particular, facial expression analysis and its related data could potentially reveal identifying information about individuals with autism; thus, researchers and potential participants (or the guardians of those individuals) are interested in ensuring that the data collected for academic research do not allow the disclosure of identifiable facial data. Therefore, our research team had to construct its own facial expression dataset specifically for the autism assessment to validate the effectiveness of the proposed method.

The construction of the database with images of children was approved by the Institutional Review Board of Central China Normal University, Ethic Committee (protocol code CCNU-IRB-202312043b and date of approval 2023.12.18).

Participants. A total of 81 participants were recruited for the data with the consent of their guardians. We took meticulous steps to ensure that the rights and well-being of the children and their legal guardians were protected. Specifically, we obtained written informed consent from the legal guardians (typically the parents or legal representatives) of the children whose images were included in the database. During the consent process, we explained the purpose, methods, potential risks, and the rights of the guardians and children involved in the study in clear and understandable language. We also provided sufficient time for the guardians to read and understand the consent form and invited them to ask any questions or express any concerns they might have. Only after the guardians fully understood and agreed to participate did we proceed with the data collection and usage.

They were aged between 4 and 6 years old and included 40 typically developing (TD) participants (mean age: 5.2 years; variance: 8 months) and 41 participants with autism (mean age: 5.0 years; variance: 14 months). The autistic children were recruited from special schools in China with the following inclusion criteria: (1) dual-blind diagnosis by two chief or associate chief pediatricians specializing in developmental behavior; (2) diagnostic criteria based on the DSM-5 published by the American Psychiatric Association; (3) age between 4 and 6 years; (4) no severe respiratory disease, schizophrenia, epilepsy, or other organic brain diseases; and (5) normal visual system development. Correspondingly, the inclusion criteria for typically developing children were as follows: (1) age-matched with the autism group, (2) no suspected or diagnosed mental disorders and/or other developmental delays or learning disabilities, and (3) normal visual system development.

Stimulus Materials: The goal was to select film clips capable of eliciting positive emotions from the participants. The film clips were initially screened based on the following three criteria:

1. Reasonable duration. Prolonged viewing leading to fatigue can affect the subjective experience of emotions. Considering the children’s attention spans and patience, it was crucial to select clips of appropriate length;
2. Easily understandable content. The testing process required obtaining emotional information from both groups of children in a short amount of time. If the meaning of a film clip were unclear, it would affect the participants’ emotional reaction time. Therefore, the content of the clips needed to be intuitive and easy for the children to understand;
3. Effective emotion elicitation. The effectiveness of emotion elicitation was a key factor in evaluating the quality of the stimulus materials.

Based on these criteria, 20 suitable film clips were initially selected. Ten volunteers were then recruited to evaluate the emotional elicitation effect of each clip through self-report questionnaires. The ten volunteers in our study, recruited from master’s and doctoral students in child education or psychology, were carefully selected for their academic backgrounds and research experience, ensuring they were qualified to assess video emotional elicitation. The questionnaire was designed based on Gross and Levenson’s work [31], assessing the elicitation levels of six types of emotions: joy, happiness, interest, sadness, disgust, and fear. Each category was rated on a 9-point Likert scale, from 0 (none) to 8 (very strong). Higher scores indicated stronger emotional intensity elicited by the film clip. Ultimately, six clips with higher scores for positive emotion elicitation (including scenes of babies playing with their mothers or pets and some amusing awkward events) were selected. These clips were concatenated in ascending order of the self-rating scores (total duration: 94 s, 25.0 frames per second). The video’s resolution was 720 × 576 pixels. To verify the validity of the stimulus materials, this research included a pre-experiment with 30 children. The statistical analysis revealed that the selected materials exhibited significant effectiveness in eliciting the target emotions (*p* < 0.05) [32].

**Figure 4:**
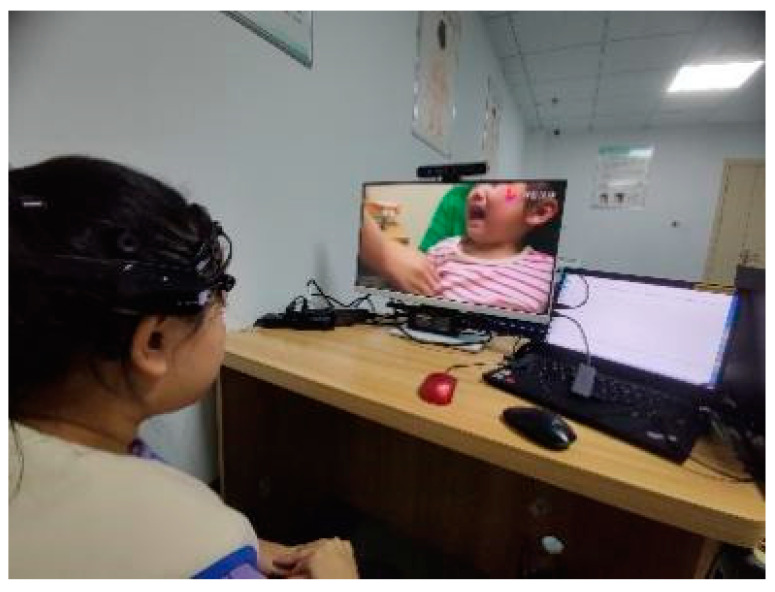
Photograph of the experimental data collection setup during the research.

Experimental Procedure: Each participant was asked to sit on a chair and adjust the seat height to face the computer screen directly. Once the participant was emotionally calm, the video playback program was started, and a camera above the display was turned on to record their behavior. To ensure the validity of the data, if the participant’s attention shifted during the experiment, they were reminded and guided. When the video playback ended, the recorded data from the camera were saved. Figure 4 depicts a photograph of the experimental data collection setup during the research.

Based on the aforementioned process, we collected facial expression videos from 81 participants, resulting in a total of 81 videos. The collected participant videos were sliced according to the duration of the video stimulus materials, with each slice downsampled to retain 200 frames (8 s), ensuring a consistent slice length. Each video was divided into six slices, with each slice consisting of 200 frames, totaling 1200 frames per video. These 1200-frame facial sequences served as the input for our algorithm.

### 4.2. Experimental Setup

This Section details the experimental setup. The facial expression intensity estimation model was trained using the method described in [26]. Considering the differences in facial expressions between children and adults, additional facial expression sequence data from typically developing children were collected to fine-tune the facial expression intensity model. The facial expression recognition model was trained using the method in [27] on the Real-World Affective Faces Database (RAF-DB) [33]. Since the RAF-DB dataset included facial expression data across different age groups, no additional fine-tuning was required. The head pose estimation model was trained using the method described in [28]. This algorithm was trained using unsupervised methods, offering high accuracy and versatility without the need for additional fine-tuning.

All binary classifiers in this paper were implemented using fully connected layers, followed by a SoftMax activation function, and the model training was supervised using binary cross-entropy loss. In the fine-grained analysis, the learning rate for training the LSTM neural network was set to $1e^{-5}$, with a batch size of 2 and 200 iterations per batch. These parameter settings are based on engineering experience and conventions established in similar works [34].

The accuracy of the proposed method was estimated using three-fold cross-validation, a technique commonly employed in facial expression analysis [35]. Specifically, all samples were evenly divided into three groups, with each group containing 27 samples. The number of participants with autism and typically developing participants in each group was as balanced as possible. Two groups were used to train the model, and the remaining group was used to test it. This experiment was repeated three times to ensure that all groups were tested once in turn, and the average of the results was calculated. The average accuracy under different experimental settings was compared in the ablation study.

### 4.3. Data Analysis

This Section describes the significance analysis of differences in coarse-grained features using IBM SPSS Statistics version 28. According to Section 3.2, 18 features were extracted from the coarse-grained analysis. These 18 features were defined in Table 1:

In this study, a one-way analysis of variance (ANOVA) was conducted on the above 18 features to explore whether there were differences among the labels. All data were divided into two groups based on whether the participant was an autistic individual. For any feature $V_{k}$, the following hypotheses were established:

**H_0_:**  *Null hypothesis: There is no difference in the labeled feature V_k_ between the two groups.*

**H_1_:**  *Alternative hypothesis: There is a significant difference in the labeled feature V_k_ between the two groups.*

The significance level α was set to 0.05. If the significance p<α, the null hypothesis $H_{0}$ was rejected, indicating that the feature was statistically significant. The specific analysis results are detailed in Table 2.

Based on the data in the Table, it can be observed that the vast majority of the coarse-grained features exhibited significant differences (*p* < 0.05), demonstrating the rationality and effectiveness of the selected feature indicators in this study. Here, we only list the features that did not show significant differences: *V*_3_ (*p* = 0.446), *V*_6_ (*p* = 0.139), *V*_7_ (*p* = 0.117), and *V*_9_ (*p* = 0.181). It is worth noting that *V*_3_, *V*_6_, and *V*_9_ are all facial expression intensity features. The main reason for their lack of significance may be that the preceding video clips had a relatively weak effect on emotional arousal, resulting in the facial expression intensity of some typically developing children not reaching the activation level. However, features such as *V*_12_ (*p* = 0.006), *V*_15_ (*p* = 0.003), and *V*_18_ (*p* = 0.042) were significant, indicating that as the emotional elicitation effect of the video clips increased, the cumulative effect of the emotions built up, and the emotional intensity of the typically developing children generally reached the activation level, making the differences between them and the autistic individuals more apparent.

As for *V*_7_, which was the head pose feature of segment 3, its lack of a significant difference may be due to the shorter attention span of autistic individuals. They usually had difficulty concentrating at the beginning of a video, showed a brief period of focused attention in the middle of the video after being reminded by the guide, and some individuals lost attention again toward the end of the video. Since the autistic individuals showed concentrated attention during segment 3, which was similar to the attention performance of typically developing children, the significant difference between the two groups was relatively small.

### 4.4. Ablation Study

To clarify the specific role of each module in the proposed method, this Section describes a comparison of the experimental results under different settings through an ablation study. In Experiment 1, we used the coarse-grained analysis module proposed in Section 3.2 to extract 18-dimensional coarse-grained features, which were then input into a binary classifier to obtain the corresponding recognition results. In Experiment 2, we used the fine-grained analysis module introduced in Section 3.3 to extract 6-dimensional fine-grained features, which were also input into a binary classifier to obtain the corresponding recognition results. In Experiment 3, we combined the 24-dimensional features extracted from both the coarse- and fine-grained analyses and input them into a classifier to obtain more comprehensive recognition results. Finally, in Experiment 4, we further incorporated the attention module proposed in Section 3.4 into Experiment 3, where the weighted fused features were input into a binary classifier to obtain the final recognition results. To ensure the reliability of the experimental results, we used three-fold cross-validation and calculated the average accuracy of the three folds. The specific experimental results are detailed in Table 3.

In Experiment 3, the average classification accuracy reached 85.07%, which was an improvement of 4.82% and 3.57% compared to Experiment 1 and Experiment 2, respectively. This significant improvement validates the complementarity of the coarse- and fine-grained analyses mentioned in Section 2. Specifically, the coarse-grained analysis focused on the characteristics of autism in terms of facial expression intensity and social consistency, while the fine-grained analysis revealed the difficulty of understanding the facial expressions of individuals with autism.

The average accuracy of Experiment 4 reached 88.74%, an improvement of 3.67% over Experiment 3. This result indicates that the attention mechanism played a significant role in the feature fusion. As mentioned in Section 4.3, some features may not show significant differences, and the application of the attention mechanism can effectively eliminate the interference of these nonsignificant features on the classification results. Additionally, when different features are combined, there may be conflicts or redundancies among them, and the attention mechanism can effectively address these issues.

### 4.5. Visualization Analysis

To further explain the results of the ablation experiments in Section 4.4, this Section presents a visualization of the attention weights. It is important to note that attention weights were obtained through data training and thus may vary during the model’s training process. Therefore, we selected the attention weights after the classification accuracy stabilized in one of the cross-validation folds for demonstration. As shown in Table 4, each row represents the attention weights of the four features corresponding to a slice (including one fine-grained feature and three coarse-grained features).

From the visualization of the attention weights, it can be seen that out of the 24 feature indicators, 11 were retained with weights of 1, while the remaining 13 had weights of 0, indicating that they did not contribute to the final classification. Compared to the univariate significance analysis in Section 4.3, attention visualization focused more on the contribution of feature combinations to classification. Therefore, the attention weights of certain features may differ from the significance analysis results. For example, in the significance analysis, the expression intensity features of slices 1–3 did not show significance, and their attention weights were also 0, indicating consistency between the two methods for these features. However, eight other features had weights set to 0, suggesting that they may not have been redundant with other features. Notably, although the head pose feature of slice 3 was not significant in the univariate ANOVA, its attention weight was 1, indicating that it still contributed significantly to the classification when combined with other features. This discrepancy may have been due to differences between the current fold data and the overall data.

Next, we analyzed the different types of features by column. The fine-grained features had weights of 1 in slices 1, 3, 5, and 6, while the coarse-grained expression category features had weights of 1 in slices 2, 4, and 5. These two types of features were each only assigned a weight of 1 in slice 5, aligning with our model’s setup; fine-grained analysis was conducted only when the coarse-grained expression category analysis was inconsistent. This demonstrates the complementary nature of the fine- and coarse-grained analyses.

**Figure 5:**
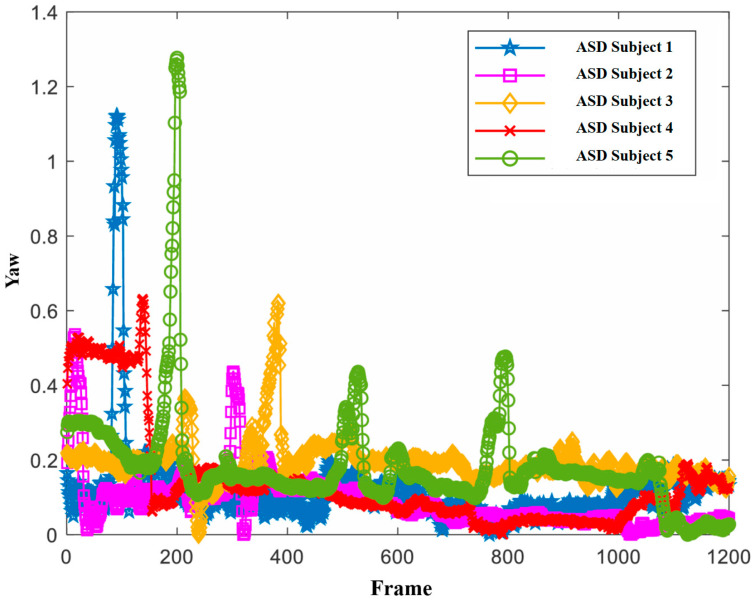
Head pose (yaw) of some of the autistic children.

**Figure 6:**
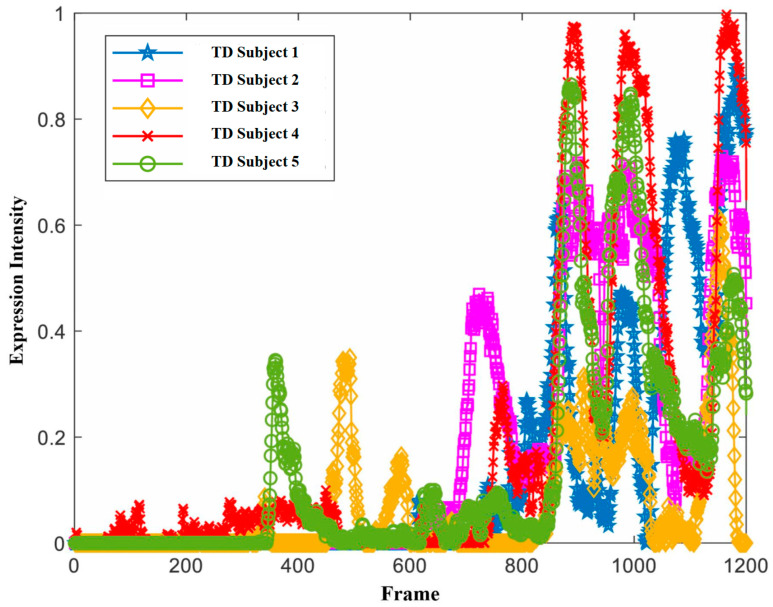
Changes in expression intensity of some typically developing children.

Additionally, we observed that the head pose features had weights of 1 in slices 1, 3, and 4, which were mostly concentrated in the first half of the viewing stimulus materials. In contrast, expressions with a weight of 1 were mostly in the latter half. This may be because the autistic children needed some time to adapt or focus their attention at the beginning of the stimulus (as shown in Figure 5), while the typically developing children could focus their attention more quickly. In the latter half, autistic children could focus their attention more quickly. In the latter half, the autistic children’s facial expressions often failed to be properly elicited, whereas the typically developing children’s facial expressions were correctly elicited and intense (as shown in Figure 6), making facial expressions more distinguishable in the latter half.

For the expression intensity features, we found that only the weight of slice 5 was 1 out of the six slices, indicating that the role of expression intensity in the combined analysis was relatively limited. This could be due to a few typically developing children not eliciting strong enough expressions when watching certain clips, as well as some autistic children eliciting other expressions with intensities reaching the pre-set threshold while watching the clips. These factors made it difficult for the model to handle outliers in the data, resulting in most expression intensity indicators not participating in the final classification.

## 5. Conclusions

In this paper, we proposed a combined coarse- and fine-grained facial behavior analysis method aimed at the intelligent identification of autism. Based on the characteristics of facial behaviors observed in individuals with autism, such as difficulty maintaining attention, reduced facial expression intensity, and a lack of social consistency in emotional expression, we constructed an interpretable facial behavior characterization model that then guides the design of a comprehensive intelligent identification system for autism. This system integrates facial intensity estimation, facial expression recognition, and head pose estimation methods to precisely extract facial behavior features from individuals with autism.

During the recognition process, the system initially generates coarse-grained analysis results through the calculation and analysis of statistical measures. Subsequently, it employs LSTM neural networks to capture temporal evolution features, yielding fine-grained analysis results. LSTM networks are particularly well-suited for capturing long-term dependencies and sequential patterns, making them ideal for analyzing the temporal evolution of facial expressions over time. This fine-grained analysis helps in detecting subtle changes and patterns that might be missed by coarser methods. Ultimately, a feature-level attention network was utilized for the adaptive fusion of all features, culminating in the final recognition outcomes.

To validate the system’s effectiveness, extensive experiments were conducted on a self-constructed dataset. The cross-validation average recognition accuracy achieved 88.74%, marking an 8.49% improvement over the recognition accuracy of a standalone coarse-grained analysis. This significant enhancement underscores the improved generalizability of facial behavior features.

Furthermore, the system’s interpretability was illuminated through ANOVA analysis and attention visualization techniques. The ANOVA analysis indicates that the majority of coarse-grained features exhibited significant differences (*p* < 0.05), confirming the rationality and effectiveness of the selected feature indicators in this study. Notably, a few features—specifically *V*_3_, *V*_6_, *V*_9_ (all related to facial expression intensity), and *V*_7_ (head pose feature during segment 3)—did not show significant differences. The insignificant results for *V*_3_, *V*_6_, and *V*_9_ could be due to the weak emotional arousal effect of the preceding video clips, which hindered some typically developing children’s facial expression intensity from reaching the activation level. Conversely, features like *V*_12_, *V*_15_, and *V*_18_ demonstrated significant differences, suggesting that as the emotional elicitation effect of the video clips intensified, the emotional intensity of typically developing children rose to the activation level, emphasizing the distinctions between them and autistic individuals. Regarding *V*_7_, the lack of significant difference may arise from the similar attention performance of autistic individuals and typically developing children at the midpoint of the video, despite the autistic group’s attention being brief. Moreover, attention visualization allowed us to visualize which features were most heavily weighted by the attention network during decision-making.

In future endeavors, we aim to expand the breadth of facial behavior characterization by incorporating additional facial features extracted by computer vision models. For instance, gaze directions can provide crucial insights into social engagement and attention patterns, while facial action units (AUs) can offer a more granular breakdown of facial movements. Furthermore, we plan to perform more extensive validation to solidify the method’s universal applicability.

## Figures and Tables

**Table 1:** Definitions of features in significance analysis of differences.

Variable	Feature	Variable	Feature	Variable	Feature
$V_{1}$	Head pose in slice 1	$V_{2}$	Expression category in slice 1	$V_{3}$	Expression intensity in slice 1
$V_{4}$	Head pose in slice 2	$V_{5}$	Expression category in slice 2	$V_{6}$	Expression intensity in slice 2
$V_{7}$	Head pose in slice 3	$V_{8}$	Expression category in slice 3	$V_{9}$	Expression intensity in slice 3
$V_{10}$	Head pose in slice 4	$V_{11}$	Expression category in slice 4	$V_{12}$	Expression intensity in slice 4
$V_{13}$	Head pose in slice 5	$V_{14}$	Expression category in slice 5	$V_{15}$	Expression intensity in slice 5
$V_{16}$	Head pose in slice 6	$V_{17}$	Expression category in slice 6	$V_{18}$	Expression intensity in slice 6

**Table 2:** Comparison of coarse-grained analysis indicators between ASD and TD (BG = Between Group, WG = Within Group, df = degrees of freedom; *p* < 0.05 indicates a significant difference).

		Sum of Squares	df	Mean Square	Statistic F	*p*
$V_{1}$	BG	1.438	1	1.438	9.468	0.003
	WG	11.995	79	0.152		
	Total	13.432	80			
$V_{2}$	BG	1.025	1	1.025	11.609	0.001
	WG	6.975	79	0.088		
	Total	8.000	80			
$V_{3}$	BG	0.141	1	0.141	0.588	0.446
	WG	18.995	79	0.240		
	Total	19.136	80			
$V_{4}$	BG	0.761	1	0.761	6.356	0.014
	WG	9.461	79	0.120		
	Total	10.222	80			
$V_{5}$	BG	1.265	1	1.265	13.329	0.000
	WG	7.500	79	0.095		
	Total	8.765	80			
$V_{6}$	BG	0.537	1	0.537	2.229	0.139
	WG	19.019	79	0.241		
	Total	19.556	80			
$V_{7}$	BG	0.292	1	0.292	2.505	0.117
	WG	9.214	79	0.117		
	Total	9.506	80			
$V_{8}$	BG	2.480	1	2.480	21.532	0.000
	WG	9.100	79	0.115		
	Total	11.580	80			
$V_{9}$	BG	0.398	1	0.398	1.823	0.181
	WG	17.256	79	0.218		
	Total	17.654	80			
$V_{10}$	BG	0.970	1	0.970	8.979	0.004
	WG	8.536	79	0.108		
	Total	9.506	80			
$V_{11}$	BG	2.856	1	2.856	21.338	0.000
	WG	10.576	79	0.134		
	Total	13.432	80			
$V_{12}$	BG	2.647	1	2.647	13.095	0.001
	WG	15.970	79	0.202		
	Total	18.617	80			
$V_{13}$	BG	0.590	1	0.590	8.032	0.006
	WG	5.805	79	0.073		
	Total	6.395	80			
$V_{14}$	BG	2.856	1	2.856	21.338	0.000
	WG	10.576	79	0.134		
	Total	13.432	80			
$V_{15}$	BG	1.973	1	1.973	9.367	0.003
	WG	16.644	79	0.211		
	Total	18.617	80			
$V_{16}$	BG	0.430	1	0.430	5.010	0.028
	WG	6.780	79	0.086		
	Total	7.210	80			
$V_{17}$	BG	2.866	1	2.866	19.387	0.000
	WG	11.677	79	0.148		
	Total	14.543	80			
$V_{18}$	BG	0.927	1	0.927	4.292	0.042
	WG	17.073	79	0.216		
	Total	18.000	80			

**Table 3:** Ablation study experimental results (√ means that the specified module is used, while × means not used).

Experiment No.	Coarse-Grained Analysis	Fine-Grained Analysis	Attention Mechanism	Classification Accuracy (%)
1	√	×	×	80.25
2	×	√	×	81.50
3	√	√	×	85.07
4	√	√	√	88.74

**Table 4:** Attention weight visualization.

Slice	Fine-Grained	Coarse-Grained		
		Head Pose	Expression Category	Expression Intensity
1	1	1	0	0
2	0	0	1	0
3	1	1	0	0
4	0	1	1	0
5	1	0	1	1
6	1	0	0	0

## Data Availability

The data presented in this study are available on request from the corresponding author due to privacy concerns and the need to protect the sensitive information of the participants.
